# Sign Language Studies with Chimpanzees in Sanctuary

**DOI:** 10.3390/ani13223486

**Published:** 2023-11-11

**Authors:** Mary Lee Jensvold, Kailie Dombrausky, Emily Collins

**Affiliations:** 1 Friends of Washoe, Ellensburg, WA 98926, USA; kdombrausky@projectchimps.org; 2 Fauna Foundation, Carignan, QC J3L7M1, Canada; emily.collins@umontreal.ca; 3 Department of Anthropology and Museum Studies, Central Washington University, Ellensburg, WA 98926, USA; 4 Project Chimps, Morganton, GA 30560, USA

**Keywords:** chimpanzee, sign language, sanctuary, ASL, vocabulary, communicative function

## Abstract

**Simple Summary:**

Chimpanzees Tatu and Loulis use signs of American Sign Language. This study presents sign use over 8 years while living at the sanctuary of the Fauna Foundation. They used a majority of the signs in their base vocabulary each year in a variety of communicative functions.

**Abstract:**

Adult chimpanzees Tatu and Loulis lived at the Fauna Foundation sanctuary. They had acquired signs of American Sign Language (ASL) while young and continued to use them as adults. Caregivers with proficiency in ASL maintained daily sign language records during interactions and passive observation. Sign checklists were records of daily vocabulary use. Sign logs were records of signed interactions with caregivers and other chimpanzees. This study reports sign use from eight years of these records. Tatu and Loulis used a majority of their base vocabularies consistently over the study period. They used signs that they had acquired decades earlier and new signs. Their utterances served a variety of communicative functions, including responses, conversational devices, requests, and descriptions. They signed to caregivers, other chimpanzees, including those who did not use signs, and to themselves privately. This indicates the importance of a stimulating and interactive environment to understand the scope of ape communication and, in particular, their use of sign language.

## 1. Introduction

Chimpanzees in sign language studies were immersed in a sign language environment [1,2,3,4,5,6,7]. All the humans and chimpanzees in these studies used American Sign Language (ASL) in interactions. This study explores two chimpanzees’ continued use of signs when living in a sanctuary with limited human signers and other non-signing chimpanzees. How robust longitudinally is the use of sign language in chimpanzees?

From the 1950s to the 1980s, there was great interest in whether chimpanzees would acquire human languages. Ethologists used the procedure called cross-fostering to study the interaction between environmental and genetic factors by having parents of one species rear the young of a different species. Researchers used this method to explore the acquisition of speech in chimpanzees [8,9]. Gardner and Gardner [1] cross-fostered the infant chimpanzee Washoe and immersed her in ASL. In a second project, the Gardners cross-fostered four more chimpanzees Moja, Pili, Tatu, and Dar [2]. In teaching ASL to the chimpanzees, caregivers imitated human parents teaching human children in human homes. All of the chimpanzees acquired and used signs in ways that paralleled human children, including the size of their vocabularies, appropriate use of sentence constituents, number of utterances, production of phrases, contextual use of signs, and sign inflection [1,10,11,12,13,14,15,16].

Adult chimpanzees Tatu and Loulis moved to the Fauna Foundation in August 2013. Their home for decades, the Chimpanzee and Human Communication Institute (CHCI) on the campus of Central Washington University (CWU), was preparing to close [17]. Tatu was 37 years old and Loulis was 35 at the time they moved. They acquired ASL as infants and juveniles and for the duration of their lives had been around humans and chimpanzees who communicated in ASL. At the Fauna Foundation, they lived among humans and chimpanzees who had no knowledge of ASL. Fauna ensured some caregivers could serve as interpreters—individuals who had cared for Tatu and Loulis at the CHCI. The interpreter caregivers also maintained written records of their sign use as they had at the CHCI. This article presents the findings from these records in two descriptive and qualitative studies. Study 1 presents vocabulary use and maintenance over 8 years at the Fauna Foundation. This provides a longitudinal picture of how the chimpanzees retained signs, the content of vocabulary, and topics of conversations. Study 2 presents the ways the chimpanzee’s utterances functioned in interactions and the illocutionary force of the utterances.

Chimpanzees gesture naturally and use the gestures of community members [18,19]. These natural gestures have meanings and are used to signal specific things. For example, the Big Loud Scratch gesture indicates where to groom. Signs, too, are gestures. Tatu and Loulis have not acquired a new capacity; instead, that capacity to gesture was shaped by the environment. The gestures of ASL were associated with specific referents and behaviors.

## 2. Method

### 2.1. Chimpanzee Biographical Information

Tatu was born at the Institute for Primate Studies (IPS) at the University of Oklahoma on 30 December 1975 and arrived at the cross-fostering laboratory at the University of Nevada-Reno at three days of age. She joined two other young chimpanzees, Moja and Pili, and all were raised like Western middle-class humans and immersed in ASL. Later infant Dar joined the project. Tatu and the other chimpanzees each had a small stable group of caregivers who only used ASL in their interactions with the chimpanzees and with each other in their presence. The chimpanzees’ days were spent in activities, routines, and daily care typical of a human child. By 13 weeks of age, Tatu had acquired 5 signs [2]. Her vocabulary continued to grow steadily from there and at 60 months she had about 135 signs in her vocabulary, and at the age of 43, she had 215 signs [13,20]. In 1981, Tatu and Dar moved to CWU. They reunited with Moja and met Washoe and Loulis.

Loulis was born at Yerkes on 10 May 1978. Washoe adopted Loulis when he was 10 months old at the Institute of Primate Studies (IPS). To determine whether he would learn signs from Washoe and other signing chimpanzees without human intervention, human caregivers restricted signing when Loulis was present except for seven specific signs: WHO, WHAT, WHERE, WHICH, WANT, SIGN, and NAME. Instead of signing, humans used vocal English to communicate in his presence. Loulis began to sign seven days after the project began. At 15 months of age, he combined signs and the development of his phrases paralleled those of cross-fostered chimpanzees and children [21]. By the time the Loulis Project ended in June 1984, Loulis was 73 months old and had 51 signs in his vocabulary. At this time, the signing restriction around Loulis ended [3,22,23]. He continued to acquire signs into adulthood, and at the age of 41, he had 78 signs in his vocabulary [3,20]. In 1980 Loulis, Washoe, and Moja moved from IPS to CWU.

As adults at the CHCI, the chimpanzees continued to sign spontaneously and interactively about activities, meals, games, and events with each other as well as with human familiars [4,5,6,20,24,25]. They had daily access to picture books, toys, clothing, and other objects, many of which were part of their lives in the cross-fostering environment and promoted natural chimpanzee behaviors such as problem solving, fine motor movement, and foraging. The CHCI facility design promoted natural chimpanzee locomotion as well. As in the cross-fostering environment, human caregivers continued to ask questions of the chimpanzees and expand on fragmentary utterances. It was during these ongoing casual signed interactions that researchers systematically explored the chimpanzees’ conversational behaviors. The chimpanzees took turns in conversation [26], initiated conversations [5], and maintained topics [16]. When human interlocutors feigned a misunderstanding, the chimpanzees adjusted their responses contingently and appropriately [24,25]. The chimpanzees’ patterns of conversation with human caregivers resemble patterns of conversation found in similar studies of human children.

In August 2013, Tatu and Loulis moved from the CHCI to the Fauna Foundation, a sanctuary in Carignan, Quebec, Canada. Fauna Foundation was founded in 1997 to provide a permanent lifetime sanctuary for chimpanzees retired from biomedical research. After Tatu and Loulis’ arrival, the sanctuary additionally welcomed chimpanzees from zoos in Quebec, which were retiring their chimpanzees. It is accredited by the Global Federation of Animal Sanctuaries (GFAS) and is a founding member sanctuary of the North American Sanctuary Alliance. The sanctuary includes indoor enclosures, outdoor enclosures, elevated tunnels, and islands. The chimpanzees lived in compatible subgroups with access to a suite of indoor and outdoor enclosures. Tatu and Loulis were the only chimpanzees at the Fauna Foundation who used signs.

### 2.2. Sign Language Records

#### 2.2.1. Sign Criteria

The form of each sign was described using a standardized system. The Place, Configuration, and Movement (PCM) system [27,28] is a description of the form of signs using the place where the sign is made, the configuration of the hand, and the movement of the hand. The PCM was described on sign logs and was an essential part of the criterion for signs to be candidates for vocabulary lists. With the exception of a few “home” signs, such as PEEKABOO and PERSON, all of the signs also appeared in standard dictionaries of ASL as described by Gardner and colleages [27]. Loulis’s PCMs appear in Fouts [3].

Gardner and Gardner [13] developed a criterion to include signs in each chimpanzee’s vocabulary list, which they described as follows: “After three separate and independent reports of a well-formed, unprompted, and appropriate observation of a new sign by three different observers, we placed the new sign on the list of candidates for reliability” (p. 225). The candidates were on the “observed” vocabulary list. Then, when an observed sign was reported for 15 consecutive days, it was moved to the “reliable” vocabulary list. This protocol was used at the CHCI as well.

#### 2.2.2. Sign Checklists and Logs

Caregivers maintained written records of signs in sign checklists and sign logs. Sign checklists were lists of each chimpanzee’s signs and provided a record of each chimpanzee’s daily vocabulary use, at least what the caregivers observed. Each day when a chimpanzee signed, a caregiver checked off that sign on the sign checklist. No matter how many times during a single day a chimpanzee made a particular sign, caregivers recorded that sign only once. The checklist documented that a particular sign was observed on a particular day; it documented its presence or absence. It did not provide a record of overall sign frequency within a day. The sign checklist form contained the list of each chimpanzee’s vocabulary items. Tatu’s base vocabulary was 215 items: 157 were reliable and 58 were observed. Loulis’s base vocabulary was 78 signs: 13 were reliable and 65 were observed.

Sign logs were a detailed record of signs in the context in which they occurred. The purpose of sign logs was to record the chimpanzees’ signing and non-signing behaviors, including chimpanzee-to-human interactions, chimpanzee-to-chimpanzee interactions, private signing, and other interesting interactions or behaviors. Sign logs documented each chimpanzee’s signed utterance during an interaction, along with the nonverbal behavior of the signer and conversation partner. They included the individual signs in each utterance, the form of each sign, the hand used to form each sign, and utterance frequency. They also included the behavioral context of each interaction (i.e., play, grooming, feeding, affinitive social, etc.), body orientation, physical location in the facility, eye gaze, vocalizations, arousal, and any other nonverbal behaviors. Last, they contained a written narrative description of the interaction [6,20]. There was a constant stream of signed interactions between Tatu and Loulis and their caregivers, so interactions of the type recorded in sign logs were ubiquitous in daily life at the sanctuary. The sign logs only represent a fraction of what actually occurred, yet still they provide a unique ethnographic record of signs, interactions, and behaviors.

On sign logs, observers recorded signed utterances into word-for-sign English because more literal translations would add words and word endings that lack signed equivalents either in the vocabularies of the chimpanzees or in ASL. This mode of transcription makes the utterances appear to be in a crude or pidgin dialect, but it is possible that equally literal word-for-word transcriptions between English and another language may appear equally crude. In this article, the examples from the logs are direct quotes of the entire narrative on the log.

#### 2.2.3. Observer Caregivers

Caregivers at the Fauna Foundation (like at the CHCI) doubled as observers. They were responsible for caregiving duties, such as cleaning, meal services, and enrichment preparation, and researcher duties, such as recording signs and behaviors during conversations and observations. They were trained in ASL, including at least one course and a demonstration of proficiency. Proficiency was demonstrated by completing a video test of each individual chimpanzee’s sign use with an accuracy of at least 85%. They also learned a chimpanzee behavioral taxonomy and demonstrated proficiency with a test score of 85% or above.

Some caregivers from the CHCI moved with Tatu and Loulis to the Fauna Foundation and continued to record sign checklists and sign logs as they had done at the CHCI. Only caregivers who had met the criterion for sign reliability completed the sign checklists. At the Fauna Foundation, this was not all of the staff—on most days, there were 1–3 signing caregivers present; however, there were some days with no signing caregiver present, which resulted in no sign checklist for that day.

## 3. Study 1. Vocabulary Production and Maintenance

The objective of Study 1 was to explore Tatu’s and Loulis’s patterns of vocabulary production at the Fauna Foundation.

### 3.1. Method

For the purpose of this study, a token is the record of a vocabulary item on the sign checklist (each day there could only be one token for a gloss). A gloss is the word for the sign and it appears in all capital letters. If the sign APPLE was recorded on two different days, that would be two tokens and one gloss. We analyzed sign checklists from 1 January 2014 to 31 December 2021. We calculated the number of checklist records. For each year, we calculated the mean number of tokens each day, the range number of tokens each day, and the total number of tokens. We calculated the number of different glosses each chimpanzee used and which glosses were used the most. We did this for all of the data and each year.

### 3.2. Results

Table 1 shows the number of days that checklists were completed each year for each chimpanzee. There was a total of 2188 checklists for Tatu and 1945 checklists for Loulis. There were some days when there was no checklist completed. These were days when there was no signing caregiver or the caregiver did not observe that particular chimpanzee signing, so the number of checklists varies each year for each chimpanzee. Table 1 shows the total number of tokens each year for each chimpanzee.

Table 1 shows for each chimpanzee the mean number of tokens that were on a checklist each day that year and the percent of their total vocabulary that the mean represents. For example, in 2014, Tatu used an average of 15.6 different tokens each day. This represents 7% of her total vocabulary. The percentage of the total vocabulary appears next to the mean in parentheses. The mean number of tokens decreased for Tatu over the years. For Loulis, it was consistent.

Table 1 shows the range number of tokens that were on checklists that year. Some days, they only used one token, but on others, they used many different tokens. For example, one day in 2014, Tatu used 58 different tokens. The range for Tatu decreased in the last few years, but for Loulis, it remained consistent. The range demonstrates that on some days, the chimpanzees used a larger percentage of their vocabulary. For example, Tatu used 21% of her vocabulary on the day she used 58 different vocabulary items. Loulis used 17% on the day he used 13 different vocabulary items.

The total number of tokens provides a rough understanding of the number of different signs the chimpanzees produced, albeit this is with the limitation that the chimpanzees may have produced a sign multiple times in one day, and it would only be scored once on the sign checklist for the day. For example, Tatu may have signed APPLE three times on a single day, but it would only have been recorded one time resulting in one token for the day.

**Figure 1:**
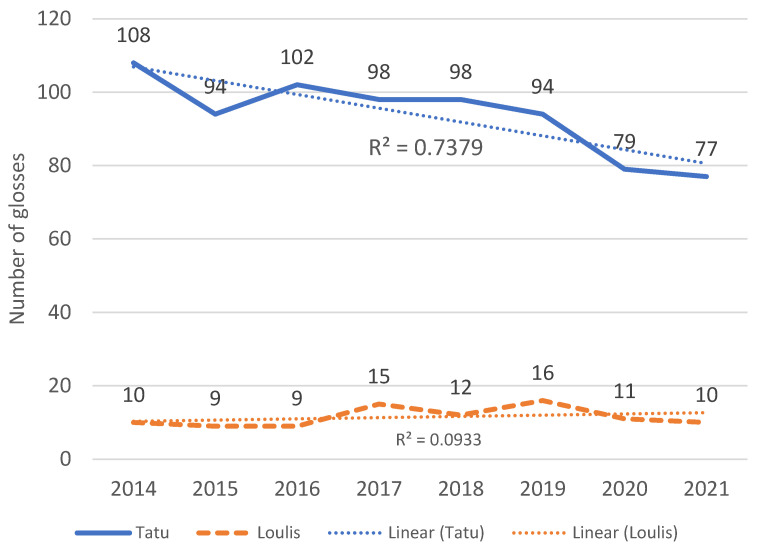
Total number of unique glosses each year and trendlines.

Figure 1 shows the total number of different glosses that each chimpanzee used each year and the trendline. For Tatu, this decreased over the study period (R^2^ = 0.73). For Loulis, it remained consistent (R^2^ = 0.09). This measure gives us an understanding of the variety of vocabulary that they used each year. The chimpanzees were free to use whatever signs they had acquired and potentially any new signs. Both Table 1 and Figure 1 show Tatu uses more signs than Loulis. She has a greater range, total, and mean number of tokens. Despite these many differences, Table 1 shows that Tatu and Loulis use a relatively similar percentage of their vocabulary. Tatu ranges from 5–7% while Loulis ranges from 4–5%. This suggests that the range, total, and mean number of tokens are a reflection of the size of the base vocabulary.

Table 2 shows the glosses that Tatu used and the total number of days the gloss was reported (tokens) for the entire study period. She used 138 different glosses. There were eight new glosses. From Tatu’s reliable list, 42 glosses (19% of her base vocabulary) never appeared in the study period. Of those that did not appear, eight were name signs of people no longer in her environment.

Table 3 shows the glosses that Loulis used and the total number of days the gloss was reported (tokens) for the entire study period. He used 20 different glosses. One was a new gloss. From Loulis’s reliable list, one gloss (0.08% of his base vocabulary) never appeared in the study period.

We also were interested in the relative ranking of the most frequently used glosses across the years as this provides a picture of content and topic of signed interactions. Table 4 shows the top five ranked glosses for Tatu and Loulis and the number of days the gloss was recorded (tokens) in that year. For Tatu, THAT appeared in the top five ranked signs every year and THERE in all but one year. DRINK appeared every year except for one and PERSON appeared every year except for two. For Loulis, CHASE and THAT ranked in the top five each year. THERE appeared every year except for one.

For each gloss, Table 4 shows its percent of the total tokens in the year. For example, in 2014, Tatu’s highest-ranked gloss was MILK, which was 5.8% of the 4401 (Table 1) total tokens for her that year. For each gloss, Table 4 also shows the percentage of days it appeared on the checklist that year. For example, Tatu signed MILK on 91% of the 283 (Table 1) days of records in 2014.

### 3.3. Discussion

#### 3.3.1. Signs in Context

While sign checklists provide information and use of vocabulary items in terms of frequency, sign logs give examples of how the signs were used in the context of interactions. We cite examples from sign logs recorded between 2013 and 2019 and quote the entire narrative portion of the logs. DRINK and THAT were frequent signs for Tatu. This example shows how both signs were used.

1 February 2018 When I returned to the chimp house following lunch, Tatu was sitting on the stairs above Room 4. I stopped in the kitchen to say hello, she signed DRINK. I glanced around but nothing stood out as truly appealing for her. I asked WHAT DRINK? She replied WATER. I followed her upstairs, where she signed THAT pointing towards the hose. I looked at the hose, and she signed WATER.

In the following example the sign DRINK functions as a verb.

30 December 2013 Today is Tatu’s birthday. When I came in this morning I told her HELLO! TODAY TATU DAY! Tatu pant-hooted while signing DRINK MILK.

Tatu often signed PERSON, which is formed with a flat hand patting the top of the head. With force, it makes noise and can function to gain attention [3,5] as in the following example.

4 November 2018 I was in the kitchen and I heard a clapping sound. I turned to look at Tatu and she signed PERSON. I verbally replied “yes”? She said THAT MEDICINE pointing to the Vitamin C bottle so I said “Oh yeah sure!” and gave her 2 Vitamin C.

The sign PERSON also refers to people, as in the following interaction. This interaction occurred after a very ill chimpanzee, Yoko, had experienced a medical procedure that required humans to enter his enclosure. Tatu had witnessed the procedure.

10 January 2014 The first day that [the medical staff] went in with Yoko they sent the rest of us out of the building. When we returned, everyone was out of Yoko’s room and Tatu very excitedly signed to me PERSON IN THERE ([the sign] there gestured to the room Yoko was in). I make [a] surprised face [and ask Tatu] IN THERE? Tatu signed PERSON THERE.

Loulis’s most popular sign was CHASE, which initiated a game of chase and appeared in interactions with humans and other chimpanzees.

4 October 2013 Binky was quadrupedal running down the long tunnel while Loulis was running parallel alongside the upstairs mezzanine windows. Binky was quadrupedal running in the tunnel, Loulis stopped at a window and signed CHASE. This is the first time I’ve seen him sign to anyone new except Sue and he mostly signs HURRY to her.

18 August 2017 Walking into the entrance this morning Lou was at Sue’s tunnel when he saw me and started signing CHASE repeatedly. I was plugging in the combination to the entrance when he started signing HURRY. We played chase for several minutes, inside and outside of the Mezzanine. When we stopped, I was by the laundry and my coffee mug was in sight. Loulis signed DRINK but I didn’t know what drink yet, and I asked WHAT DRINK? He signed THAT towards my coffee mug.

HURRY also was one of Loulis’s higher-ranked signs.

10 July 2017 I was cleaning Room 1 in the morning when Loulis approached the door to the Mezzanine that faces Room 1 downstairs. Loulis bronx cheered to get my attention. I turned and met his gaze, he signed THAT at me. I responded WHAT DO NOW? He signed DRINK so I gave him a drink from the hose. As I prepped the hose Loulis signed HURRY.

#### 3.3.2. Environmental Constraints

When Tatu and Loulis arrived at the Fauna Foundation, they encountered many new caregivers, chimpanzees, routines, and the environment. The Fauna Foundation endeavored to keep as many constants in their lives, such as food and the caregivers who came with them from the CHCI. But there were changes and we can see that in the top signs. For example, caregivers began offering crackers to Tatu in the last couple of years of the study. This is one of her favorite foods and CRACKER appeared in her top signs at that time. She received cheese often at the beginning of the study period and CHEESE ranked in the top five one year. She is lactose intolerant so caregivers stopped offering it and it dropped down in her ranking. Loulis always loves a game of chase and this sign always appeared in the top ranking. In later years, he liked receiving drinks from the hose and DRINK moved into the top ranking. Dombrausky and colleagues [29] presented the top-ranked five signs for the years 2004–2006 when Tatu and Loulis were at the CHCI. The top signs varied and included SMELL, MASK, CRACKER, BLACK, THAT, and ONION. There is little overlap with the top signs in this report except for the indexical sign THAT. During 2004–2006, Loulis’s top signs were CHASE, THAT, HURRY, GIMME, and FOOD [29]. For Loulis there is a fair amount of overlap between the earlier study and this study.

#### 3.3.3. Indexical Signs

Indexical signs are signs that include a point and have a variety of functions. For example, “Often a signer will indicate the location of an entity (a person, or a thing or a place) using a sentence such as MAN THERE, in which THERE is an index finger pointing downward toward a specific place.” (p. 75) [30]. Additionally, “indexical signs have some of the same general functions as free-standing pronouns.” (p. 277) [31]. While human signers use indexical signs grammatically and systematically, these signs are easily intelligible to both signing and non-signing individuals. The indexical signs in Tatu’s and Loulis’s vocabularies are THAT, THERE, ME, and YOU. Except for ME, these signs were consistently in the top five ranked signs for Tatu and Loulis.

Jensvold and Dombrausky [20] presented the top five ranked signs for the years 2004–2007 when Tatu and Loulis lived at the CHCI. During that time, they each only had the sign THAT in the top five rank. Tatu and Loulis perhaps adjusted to using signs that were more intelligible to all of their caregivers, signing and non-signing, at the Fauna Foundation. We must keep in mind these top-ranked signs are only a fraction of the overall vocabulary use, particularly for Tatu, so this is only one of several ways to explore how the chimpanzees used their signs.

The indexical signs, either THAT and/or THERE, always appeared in the Location subcategory. For example:

7 January 2014 Tatu sits up on scale downstairs in Jeannie’s area. She looks toward the kitchen and signs DRINK. I look around and do not see a drink. [I ask] WHERE? Tatu signs DRINK THERE. [I reply] I DO NOT UNDERSTAND. Tatu signs STUPID [I ask] WHAT YOU WANT? Tatu signs THAT, to kitchen… I still don’t know what drink she was signing about but I gave her a vitamin C tablet and she seemed happy.

Of note is the difference in how Loulis and Tatu acquired signs: Tatu from humans and Loulis from Washoe and other chimpanzees. The other cross-fostered chimpanzees also had vocabulary sizes comparable to Tatu’s; at the time of their deaths, Washoe’s was 245, Moja’s was 209, and Dar’s was 175 [20]. Despite Washoe’s large vocabulary, Loulis still had the smallest vocabulary. Loulis’s sign language exposure began at 10 months and Washoe’s also was in the latter part of her first year. But for the other cross-fosterlings, the first sign exposure was in the first days of life [10]. Thus, early exposure or ways to acquire signs may not play a role in the preservation of signs and vocabulary; instead, once acquired, they stick.

#### 3.3.4. Memory

This study spanned eight years and showed retention and memory for many signs that the chimpanzees had acquired when they were young, meaning they had remembered and used them for decades. Some signs were things that were salient and part of their daily environment, such as DRINK. Others were names of people who no longer were in the chimpanzees’ lives. One example from the logs is in regard to the chimpanzee Dar, who had been deceased for almost four years at the time of the conversation.

16 January 2018 Tatu clapped to get my attention this morning and when I looked over she signed DAR. I replied, with a very confused look, DAR? She repeated DAR, but didn’t elaborate. Maybe she dreamt about her old friend.

Bonobos and chimpanzees were trained to use lexigrams, a symbolic system, in which symbols stood for words. In formal annual vocabulary tests over 10 years, they showed retention of lexigrams. Chimpanzee Lana’s vocabulary was smaller than that of the other apes, partially because experimenters had removed some of the lexigrams from her system [32]. Remarkably, Lana showed that she remembered the lost lexigrams after 20 years without them [33]. Removing symbols is in stark contrast to gestural communication: as long as apes have their hands, they are able to make signs, and there is nothing to stop them. Because signs are natural and embodied, the chimpanzees can produce new signs as well, which we saw in this study.

Cases of signing apes with no exposure to signs provide remarkable examples of sign retention. Bodamer [34] described meeting a chimpanzee Bruno at the Laboratory for Medicine and Surgery in Primates (LEMSIP). Bruno had learned signs as a child before he lived in the laboratory. Bodamer’s meeting was after Bruno had been in the laboratory for 16 years. None of the laboratory technicians knew signs so Bruno had been without exposure all of those years. In ASL, Bodamer asked Bruno his name. Bruno replied KEY OUT (pp. 228–230). Chantek was an orangutan who used signs until his death at age 39. In the last years of his life, he used signs with his keepers at Zoo Atlanta [35]. As an infant, he acquired signs in an interactive environment at the University of Tennessee at Chattanooga with caregivers who used signs. From there, he lived in the Yerkes Regional Primate Laboratory and later Zoo Atlanta. He, like other apes, showed long-term retention of signs in a variety of social and linguistic settings. Sign language is a robust behavior in chimpanzees.

## 4. Study 2. Communicative Function

While individual signs provide information about content and topics in the chimpanzees’ utterances, their communicative function is another way to explore signed interactions. Utterances have illocutionary force, which is how they function—for example, to ask or answer a question, begin or end a conversation, make statements, agree, negate, and so forth. A conversationalist must know how to use utterances in actual interactions. They must understand the effect that the utterance will have on a partner [36,37,38]. The communicative function allows for a pragmatic analysis of an individual’s language and conversational competency. The communicative function is the intention or motive for an utterance [36]. In two studies, Dore [37,38] categorized utterances of white middle-class American 34- to 42-month-old children into seven categories (see Table 5). Though Dore developed the categories of communicative function in the 1970s, they are still relevant and used in current communicative function and language development research [39,40,41]. 

Leeds and Jensvold [6] categorized communicative functions in 1057 utterances of Tatu, Loulis, Washoe, Moja, and Dar recorded from 2000 to 2003 during unstructured interactions with each other and caregivers. They categorized utterances using seven categories and subcategories adapted from Dore [37,38]. In Leeds and Jensvold, the chimpanzees used all of the categories. The data in that study were recorded at CHCI where as stated all of the caregivers were proficient in ASL. The objective of Study 2 was to replicate the method of Leeds and Jensvold [6] and investigate the communicative functions of Tatu’s and Loulis’s utterances in the new linguistic environment at the Fauna Foundation.

### 4.1. Method

The first six months after Tatu and Loulis moved to the Fauna Foundation were particularly interesting times since they were meeting new chimpanzees and caregivers and experiencing new routines. Study 2 analyzed the distribution of communicative function categories from sign logs recorded at the Fauna Foundation from August 2013 to February 2014, which covered this time. Coders categorized each utterance on the sign log into categories (Requests, Responses, Descriptions, Statements, Conversational Devices, Performatives, Uninterpretable, and Private Sign) and subcategories using the definitions in Table 5. They also used operational features, which were the semantic content of the utterance, the accompanying nonverbal behavior of the chimpanzees, the behavioral and social context of the interaction, and the behavior of the partner [6]. This information appeared on sign logs. A single sign log contained one or more utterances. A single utterance could contain one or more signs, yet the entire utterance was classified. The coder used all information available on the log to make the categorical determination. Some utterances met the definition of multiple categories.

To determine interobserver agreement, the two coders categorized all of the utterances independently. Interobserver agreement was 80%. After independent coding and calculation of interobserver agreement, the coders discussed disagreements and decided on a category and subcategory.

### 4.2. Results

#### 4.2.1. Categories of Communicative Function

There were 131 utterances in the logs. Table 5 shows the number of utterances in each category and subcategory. The chimpanzees used all seven categories. Response was the most frequent category at 31.3%. In Leeds and Jensvold [6], it was also the most frequent category at 35%. For hearing children, it was 18.5% [37,38], and for deaf children, it was 10.5% [42]. Caregivers ask many questions; it is the nature of their job, which includes offering choices of foods and activities as in the following example:

20 November 2013 Tatu ate her multivitamin and soymilk cup. She asked for MILK. I replied MILK FINISHED. YOU WANT FRUIT? Tatu signed FRUIT. I signed APPLE BANANA PEACH WHICH? Tatu signed PEAR.

In this example, the first response is the subcategory Yes-No Answer and the second is Wh-Answer. Other answers to questions were about the environment as in the following interaction about a maintenance man, M, whom Tatu knew.

6 February 2014 Tatu was sitting on the ground in Jeannie’s area. I sat down next to her and asked M if he wanted to sit with us. I asked Tatu WHO THAT? Tatu signed FRIEND. I signed BOY GIRL WHICH? Tatu signed BOY. I signed YOU FUNNY!

In the above example, the responses are in the Wh-Answer subcategory. The chimpanzees used all of the subcategories in the Response category reflecting the variety of question types from caregivers and the variety of ways to answer.

Conversational Device was the second most frequently used category at 27.4%. In Leeds and Jensvold [6], this was the least frequently used category at 0.9%. For deaf children, it was 18.2% [42], which was the third most frequent; however, for hearing children, this category was the least frequent [37,38]. This category serves a pragmatic function in initiating and ending conversations as in the subcategory Boundary Marker. The following is an example of that:

10 November 2013 Loulis sits up on ground in upstairs apartment. Binky and Maya [were] in Back 1. They’re sitting near the caging. Loulis signed HURRY towards Binky and Maya. Binky did not respond. Maya did not respond.

Loulis’s attempts to initiate an interaction with the sign HURRY fail. The following interaction is an example of the subcategory Accompaniment:

12 February 2014 Loulis is clinging on the cage in Room 1, [Caregiver T] is getting the [dinner] trolleys ready with tonight’s vegetables. Loulis begins to breathy-pant and orients himself at Spock. Loulis signs HURRY to Spock while climbing on the caging toward Spock. Spock is sitting up on the bench in Room 1 looking towards Loulis. They both breathy-pant and Loulis approaches Spock. They embrace, hug and open mouth kiss each other. After, Loulis climbs down onto the ground and Spock breathy-pants and looks toward the kitchen.

Breathy pants are an indicator of the excitement of the anticipation of a meal. The sign HURRY accompanies the general shared excitement.

Request was the third most frequently used category at 25.9% of utterances. In Leeds and Jensvold [6], it was 12.5%. For human children, it ranged from 20 to 50% [37,38,42,43]. Children make more requests in a structured environment [44], and a sanctuary is a structured setting in which the chimpanzees cannot help themselves to goods and services, and activities occur in a routine. The chimpanzees who sign easily can make specific and extra requests as in the following example:

7 October 2013 I gave [Tatu and Loulis] each a tofu dog around 2 pm today. A few minutes later, Tatu was lying in the hammock outside and asked MORE MEAT. I signed YES! Wait there…” I went to get another tofu dog (plain, by the way—no bun or mustard or anything).

Requests also could serve as requests for action. For example:

26 October 2013 Tatu goes into apartment. Spock enters [an adjacent room] Back 1. Tatu approaches and bounces towards Spock. Spock sits. Tatu sits beside Spock. Tatu signs GROOM looking toward Spock.

Performatives appeared in 12.9% of utterances. In Leeds and Jensvold [6], it was 7.9%. For hearing children, it was 10.8% [37,38], and for deaf children 5.7% [42]. In Study 2, most of the utterances were in the subcategories of Game Markers and Reassurance. It was during this time that Tatu and Loulis were experiencing the first introductions to new chimpanzees, Spock and Sue Ellen. They were meeting other chimpanzees through wire fencing as well. This may have affected the high number of utterances in the Reassurance subcategory since chimpanzee introductions are highly arousing situations. A typical example of Reassurance follows in the interaction between Loulis and chimpanzees in an enclosure adjacent to him:

26 December 2013 Toby [another chimpanzee] and Loulis had been displaying on opposite sides of the upstairs (Lou on Mezzanine side, Toby on Jean’s side). Toby started by raking a metal bowl on the floor. Lou did the same. Toby banged on the door separating them. Loulis clung to the cage on the Mezzanine side. When Toby took a few steps back, Loulis banged on the door. Rachel [another chimpanzee in Toby’s enclosure] came over and hit the door a few times. Loulis climbed higher on the caging and signed HURRY to Toby. Toby came over and sat down in front of Rachel.

Descriptions appeared in 11.4% of the utterances. In Leeds and Jensvold [6], it was 19.0%. For hearing children, it was 22.3% [37,38], and for deaf 28.6% [42]. This category includes labeling and describing aspects of the environment. Labeling occurs in the following example of the subcategory Identification:

18 February 2014 I brought Tatu a drink and told her QUIET SODAPOP IN THERE (it was Nestea Zero). Tatu takes a few sips. I verbally ask Spock “Do you want some of this drink?” Tatu looks at Spock and signs SODAPOP.

Location is another subcategory in Description in which the utterance describes a location or direction. The following interaction occurred during the cleaning routine. Tatu’s first three utterances indicate locations. Then, the conversation moves to Caregiver K (the observer refers to her as KEY GIRL), who is responsible for unlocking enclosures during the cleaning process.

4 February 2014 Tatu was sitting up on the bench upstairs Mezzanine. Tatu signed LOTION THERE. I gave her some lotion. Tatu signed IN. I asked IN WHERE? Tatu signed THERE (towards Room 1). I signed IN SOON KEY GIRL THERE CLEAN (Caregiver K). Tatu signed KEY. I signed YES THAT KEY GIRL. Tatu signed HURRY KEY

Statements appeared in 3.8% of utterances. In Leeds and Jensvold [6], it was 3.9%. For hearing children, it was 13.8% [37,38], and for deaf, it was 10.3% [42]. The highest subcategory was Internal Report. These utterances were interesting in that they refer to physical sensations such as hunger as in the following example:

11 November 2013 Tatu was sitting beside Jeannie’s door upstairs in the Mezzanine. Tatu signed PEACH. I signed “Sorry can’t. You hungry?” Tatu signed HUNGRY. I signed “Want vegetable?” Tatu signed VEGETABLE. I brought her (and Chance [another chimpanzee] who was sitting nearby) a cucumber.

Internal reports also refer to sentiments. SORRY was in several of the interactions in this study. For example:

18 October 2013 The day after they first met, Tatu and Spock were playing tickle and wrestle in Jeannie’s front Room 1. Chance [another chimpanzee in an adjacent enclosure] came into Room 5 and began screaming. Tatu sat up, startled. She looked at Chance and signed SORRY.

This interaction was coded with two codes. The sign SORRY was both a Statement: Internal Report and a Conversational Device: Politeness Marker.

#### 4.2.2. Interaction Partner

For the combined chimpanzees, Table 6 shows the distribution of communicative function categories in interactions with humans (C-H) or other chimpanzees (C-C). Using a chi-square goodness of fit test we compared the distribution of the C-H values to the C-C distribution of proportions and calculated standardized residuals. The distribution between the two is significantly different *X*^2^ (6, N = 88) = 4042.78, *p* < 0.0001. The Request category was significantly greater in C-H than in C-C (z = +3.33). The Response category was significantly greater in C-H than in C-C (z = +59.64). The Descriptions category was significantly greater in C-H than in C-C (z = +20.44). The Conversational Device category was significantly lower in C-H than in C-C (z = −5.26). Performative was significantly lower in C-H than in C-C (z = −4.48).

### 4.3. Discussion

#### 4.3.1. Partner and Communicative Function

The communicative functions varied with the partner, whether human or chimpanzee. In C-C interactions, there were no utterances in the Response category. That category required a previous utterance from the partner. Leeds and Jensvold [6] also found the same result. In the earlier study, all of the chimpanzee partners communicated with signs. Thus, this finding was not necessarily a result of the signing ability of the partner. In the conversations with other chimpanzees, the Conversational Devices category was frequent. It was often used to initiate interactions with other chimpanzees and a reflection of the varied and novel social environment. Performative was also high in C-C interactions, reflecting the types of interactions between conspecifics: grooming, greeting, reassurance, and play.

Rivas [45] claimed that Request was the most frequent communicative intention (86%) in an analysis of C-H interactions with the chimpanzees at the CHCI. Our results are quite different. In this study, Requests were 27% for C-H but only 13% for C-C, and it was never the most frequent category. Rivas had fewer and different categories than this study. Some of the corpora in Rivas were video recorded under highly scripted conditions and others during meal time interactions. In contrast, this study captured casual interactions in a variety of settings with a variety of partners. These differences between findings point to the problems with relying completely on C-H interactions to understand communicative function in the signing chimpanzees.

The logs sampled in this study span the first six months that Tatu and Loulis were at the Fauna Foundation. Tatu had never before met non-signing chimpanzees, and for Loulis, his only exposure to non-signing chimpanzees was during his infancy at Yerkes. As time transpired, Tatu and Loulis signed less often to the other chimpanzees, but they did not stop completely [20]. Jensvold and Dombrausky [20] presented the individual recipients of C-C interactions logs from 1 September 2013 to 31 August 2018. There were 80 C-C utterances. Both Tatu and Loulis most often signed with Spock who was one of the first chimpanzees introduced to their group. Sue Ellen also was one of the first chimpanzees introduced to their group yet there were no recorded C-C utterances toward her. Tatu and Loulis also often signed to chimpanzees in adjacent enclosures. This indicates sociality between separate social groups in captivity. This supports other research that shows interactions between neighboring groups of captive chimpanzees can be a source of social stimulation [46,47,48]. The logs provide some insight into the nature of introductions between chimpanzees and their perceptions of the individuals around them.

#### 4.3.2. Uninterpretable Utterances

Coders used the Uninterpretable category for 6.8% of the utterances, which was lower than Leeds and Jensvold [6] at 20.8%. The interobserver reliability for Leeds and Jensvold was 91.56%, which was higher than in this study. Communicative function is difficult to determine. We cannot know what the chimpanzees are thinking, but communicative function is a way to objectively determine the intent of the utterances. Both studies had a higher number of Uninterpretable in the C-C interactions than in the C-H interactions, indicating that perhaps without the human perspective, it is more difficult to understand the intention behind utterances.

#### 4.3.3. Private Signs

Private signing is when a chimpanzee signs with no indication of interaction. Private signing is well documented in the signing chimpanzees [49,50] and a future study could explore private signing in logs recorded outside the time frame of Study 2. For example:

26 July 2014 I was sitting looking for Tatu in the upstairs mezzanine when I poked my head out of the window and saw her in the beginning of Lou’s tunnel. She was sitting there all alone and she signed COFFEE. She must have thought it was a good time for coffee. She didn’t notice me at the window either.

13 May 2014 Tatu sat up in Lou’s tunnel and held up a mirror with her left hand. She looked at her reflection and signed EARRING 3 separate times.

The following example was from Study 2.

28 January 2014 Tatu was lying down on a pile of blankets while I groomed her side. She signed Tatu CARROT LOTION. [Then again] Tatu CARROT LOTION CARROT. It looked like she was just ‘rhyming’.

When the Place, Configuration, or Handshape has the same form between two signs, that is analogous to spoken rhyming [31]. The place for both CARROT and LOTION is on the palm and pinkie finger side of the hand. The movement, for both signs, pulls across the palm toward the pinkie edge. The configuration is what varies, CARROT is an extended thumb and LOTION is a pincer hand. This supports previous reports of rhyming in private signing with Moja [50].

#### 4.3.4. Sign Modulation

Sign logs contained information about sign modulations and nonverbal behaviors. Signers of ASL modulate signs grammatically and systematically to change the meaning of signs. These can occur in, for example, movement such as faster or slower; placement such as on an object or addressee; and in facial expressions. For example, lowered eyebrows and held signs mark Wh-questions while raised eyebrows mark Yes-No questions [51,52]. The chimpanzees use these markers as well [27]. Place changes occurred, for example, on the addressee, an object, or the body [15]. Tatu modified action signs such as GO directionally in response to Where questions [16] but not in response to “What that?” question. In this study, caregivers recorded notes on modulation on the sign logs both in the narrative and in the description of the sign’s PCM. Coders used the description of the sign modulation to determine coding. In the example of Location on 4 February 2014 (see Section 4.2.1), there are numerous examples of directional inflection in Tatu’s signs of THERE.

Coders also used notes on facial expressions, vocalizations, body posture, and other gestures that appeared on the log. For example

25 December 2013 Loulis was sitting in the Room 1/Apartment tunnel area (in Sue Ellen’s bed). [Caregiver G] had just handed out Christmas gift bags and everyone was really excited. Loulis was breathy panting and also fear grimacing. Spock was sitting on the upstairs platform of Back 1. Spock laid down on the platform with his face oriented towards Loulis. They breathy panted [oriented toward] each other for a few minutes, and then Loulis signed HURRY [with both hands] to Spock. They continued breathy panting at each other.

Reassurance is a context that occurs in high-arousal situations when two chimpanzees come together and share in the excitement and also calm each other [53]. In the description, it is an exciting situation where a caregiver is distributing Christmas gifts. Loulis had behaviors associated with high arousal—breathy panting is a repeated audible exhale that occurs in greeting and reassurance seeking. His face is a fear grimace, also known as a full open grin. It does not necessarily imply fear (although it can indicate that emotion), but it indicates high arousal and can happen in exciting moments [53]. Loulis is seeking reassurance from Spock. Notes on the PCM indicate Loulis signed HURRY with two hands, which is a modulation that adds emphasis to the sign [54,55]. Spock breathy pants in unison with Loulis, which provides solidarity and reassurance. These notes would be useful to the coder to make the determination of the Performative category and Reassurance subcategory.

There is much information on the logs that remains to be explored in future studies that would utilize the details of signs and nonverbal behaviors.

## 5. General Discussion

### 5.1. Signs in Sanctuary

Tatu and Loulis continue to use signs as a modality of communication throughout their day-to-day lives despite years of a different linguistic environment. Our results offer some insight into the social interactions experienced by Tatu and Loulis, and ways in which these two individuals use signs to exchange information with others across varying contexts. Language is more than just words and grammar. It includes knowing what to say and to who [41]. The communicative function is a way to operationalize that and to understand the meaning and purpose of utterances and their social function. Applying this method to the chimpanzees’ utterances provides insight into their social lives and cognition in the sanctuary setting.

The chimpanzees at the Fauna Foundation receive a variety of activities each day. Environmental enrichment includes toys, clothes, accessories such as hair clips and necklaces, crayons, paint, blankets, food puzzles, and forages. They have access to a variety of spaces inside and outside, and their daily routines include shifting between spaces for cleaning purposes. They receive a varied meal plan with a variety of drinks and food, such as fruits, vegetables, and grains that may be cooked, raw, frozen, or dried. Most importantly they have caregivers who understand and use chimpanzee behaviors in interactions. Caregivers attend to chimpanzees‘ individual needs and listen. Tatu and Loulis interject signs into these activities. Signs are ubiquitous in the daily interactions with the signing caregivers. The method of recording ad libitum for sign logs and the records of sign checklists created a picture of interactions in this relaxed natural setting rather than structured experimental trials.

The logs reflect the casual nature of the sanctuary. Conversations were about other individuals, relationships, and sentiments. The logs reflect the ethos of the sanctuary and the personal daily relationships between the caregivers and chimpanzees; the connection is captured in the logs and signs.

### 5.2. Individual Differences and Generalizability

Tatu and Loulis show individual differences in vocabulary size and use. Previous studies have found individual differences in preferences and interaction styles, for example, between all of the signing chimpanzees [5,56]. This study showed differences in the top-ranked signs—Tatu’s included more foods while Loulis signed more about social activities; at the same time, there was overlap in indexical signs. Temperament and developmental histories account for some differences between individuals [32,57]. Differences in developmental histories contribute to differences in cognitive processes among humans [58,59], and there is evidence to suggest this is true of nonhumans as well [60,61]. The unique histories of Tatu and Loulis mean that these results are difficult to generalize to other chimpanzees, and at the same time, they do provide some insight into the mental processes of this species. At the very least, this information can be used to infer that non-signing chimpanzees have internal feelings, preferences for foods, activities, places, and an awareness of the social environment.

This research raises questions about the need or value of captive chimpanzees learning signs to communicate with caregivers. Acquiring signs was easy for the young chimpanzees Washoe, Moja, Tatu, Dar, and Loulis. The adult chimpanzees at the Fauna Foundation initially observed with great interest the signed interactions between Tatu and Loulis and their caregivers. Despite this, we never observed the other chimpanzees signing. In humans, there is some evidence to support a critical period for some aspects of language [62]. It might be more difficult for adult chimpanzees to acquire signs than for younger chimpanzees. With breeding prohibited in accredited sanctuaries, the North American chimpanzee population is largely older individuals so researchers might instead invest energy in understanding natural chimpanzee behaviors to improve communication and welfare.

## 6. Conclusions

Tatu’s and Loulis’s use of ASL is a robust and flexible aspect of their communication. It has persisted for decades through different living environments, all of which have been ones that foster signed communication including the sanctuary setting. They use the signs flexibly with a diversity of communicative functions in interactions with humans and chimpanzees. Chimpanzees and other apes naturally use gestures in the wild, acquiring them from community members [18,19]. This shows a biological predisposition as well as the influence of the gestural community both in the wild and in captivity.

## Figures and Tables

**Table 1:** Descriptive statistics of token production for each year.

		Tatu				Loulis		
	Number of Checklists	Range	*M* (%)	Total Tokens	Number of Checklists	Range	*M* (%)	Total Tokens
2014	283	1–58	15.60 (7)	4401	254	1–10	3.96 (5)	1005
2015	257	1–33	12.48 (6)	3208	189	1–9	3.84 (4)	736
2016	238	1–32	14.47 (7)	3446	181	1–12	4.11 (5)	744
2017	270	1–46	14.19 (6)	3832	234	1–12	4.39 (5)	1033
2018	303	1–32	12.06 (6)	3666	343	1–12	3.73 (4)	1279
2019	312	1–26	11.88 (6)	3707	260	1–8	3.61 (5)	938
2020	273	1–24	10.79 (5)	2923	237	1–9	3.93 (5)	928
2021	252	2–21	10.51 (5)	2650	247	1–13	4.02 (5)	949

Note: *M* is the mean number of glosses recorded on a checklist in a day during that year. In parentheses is the percent of total vocabulary for the mean (Tatu *M*/215; Loulis *M*/78).

**Table 2:** Tatu’s glosses and the number of total tokens during the study period.

DRINK	1746	GRASS	201	GIRL	32	VEGETABLE	3
THAT	1531	MEDICINE	195	CANDY	31	GLASS	2
PERSON	1509	SODAPOP	189	SWALLOW	30	MARY LEE J	2
THERE	1422	FOOD/EAT	171	UP	25	METAL	2
YOU	1307	CORN	145	BOY	22	SHIT	2
MILK	1298	TEA	137	OUT	21	TIME	2
GO	1213	FRUIT	131	POTTY	20	WRISTWATCH	2
HURRY	1065	POTATO	123	COW	19	*BAG*	1
IN/ENTER	954	TATU	116	PEA/BEAN	19	COLOR	1
CRACKER	920	BIRD	112	CAT	18	DEBBI F.	1
BANANA	871	FLOWER	112	TICKLE	18	DIAPER	1
SMELL	859	BREAD	98	ME	14	DIRTY	1
APPLE	820	HURT	96	BUG	12	FUNNY	1
BLACK	810	GRAPES	92	PLANT	12	GLOVE	1
MASK	723	FRIEND	82	BRUSH	11	GREEN	1
GIMME	680	LAUGH	80	HEAR/LISTEN	10	HORSE	1
CHEESE	623	TOOTHBRUSH	77	PLEASE	10	HUG	1
CARROT	602	ICE CREAM	76	SHOE	10	NO	1
MEAT	577	BLANKET	69	DOG	9	*NOSE*	1
COFFEE	504	PEAR	69	EARRING	8	PEN/WRITE	1
MORE	434	WATER	65	GOOD	8	ROGER F.	1
NUT	431	POPCORN	61	HOT	8	*SANTA*	1
CEREAL	387	PAINT	55	WHITE	8	*SICK*	1
RED	346	QUIET	52	KISS	7	SLEEP	1
TREE	304	SORRY	49	DAR	6	*SOON*	1
OIL/LOTION	300	SLICE	48	GARBAGE	6	*STUCK*	1
GROOM	274	ORANGE	47	TOOTHPASTE	6	SURPRISE	1
CHASE	271	KEY	45	BED	5	THINK	1
PEACH	264	SANDWICH	44	HAIR	5	*THIRSTY*	1
CLEAN	264	BABY	42	CRY	4	TOMATO	1
ONION	254	COME	42	MINE/MY	4	WHO	1
SWEET	250	GUM	42	CAN’T	3	*YELLOW*	1
ICE/COLD	240	COOKIE	40	HUNGRY	3		
BERRY	220	RICE	37	RADIO	3		
LIPSTICK	205	CLOTHES	34	STUPID	3		

Note. New signs are italicized.

**Table 3:** Loulis’s glosses and the number of total tokens during the study period.

CHASE	1789	GIMME	91	PERSON	2
THAT	1683	TICKLE	32	MASK	2
THERE	1211	COME	13	SWALLOW	2
HURRY	1191	GOOD	13	GO	1
YOU	740	TOOTHBRUSH	8	HUG	1
DRINK	522	KISS	6	NO	1
FOOD/EAT	346	*CORN*	3		

Note. New signs are italicized.

**Table 4:** Top five ranked glosses each year.

			Tatu				Loulis	
		Total Tokens	% Total Tokens	% Total Days		Total Tokens	% Total Tokens	% Total Days
2014	MILK	258	5.86	91.17	CHASE	233	23.18	91.73
	DRINK	235	5.33	83.04	HURRY	196	19.50	77.17
	YOU	197	4.47	69.61	THAT	193	19.20	75.98
	THAT	191	4.33	67.49	YOU	141	14.02	55.51
	THERE	180	4.09	63.60	THERE	76	7.56	29.92
2015	MILK	210	6.54	81.71	CHASE	170	23.09	89.95
	CHEESE	182	5.67	70.82	THAT	153	20.78	80.95
	PERSON	158	4.92	61.48	YOU	123	16.71	65.08
	THAT	157	4.89	61.09	THERE	119	16.16	62.96
	YOU	154	4.80	59.92	HURRY	100	13.58	52.91
2016	THAT	180	5.22	75.63	CHASE	158	21.23	87.29
	THERE	171	4.96	71.85	THAT	151	20.29	83.43
	DRINK	169	4.90	71.01	THERE	124	16.66	68.51
	APPLE	154	4.46	64.71	HURRY	106	14.24	58.56
	YOU	140	4.06	58.82	YOU	94	12.63	51.93
2017	THAT	212	5.53	80.20	CHASE	277	26.81	96.60
	THERE	204	5.32	66.34	THAT	203	19.65	86.38
	DRINK	194	5.06	62.38	THERE	160	15.48	68.09
	PERSON	191	4.98	55.45	HURRY	150	14.52	63.83
	YOU	187	4.88	55.45	YOU	116	11.22	49.36
2018	DRINK	243	6.62	70.85	CHASE	317	24.78	92.42
	THAT	201	5.48	58.6	THAT	298	23.29	86.88
	PERSON	189	5.15	55.10	HURRY	194	15.16	56.56
	THERE	168	4.58	48.98	THERE	188	14.69	54.81
	YOU	168	4.58	48.98	YOU	124	9.69	36.15
2019	DRINK	274	7.39	87.82	CHASE	234	24.94	90.00
	PERSON	246	6.63	78.85	THAT	220	23.45	84.62
	THAT	227	6.12	72.76	THERE	153	16.31	58.85
	THERE	205	5.53	65.71	HURRY	145	15.45	55.77
	MILK	189	5.09	60.58	DRINK	68	7.24	26.15
2020	DRINK	245	8.38	89.74	THAT	226	24.35	95.36
	PERSON	228	7.80	83.52	CHASE	219	23.59	92.41
	THAT	194	6.63	71.06	THERE	188	20.25	79.32
	THERE	187	6.39	68.50	HURRY	145	15.62	61.18
	GO	174	5.95	63.74	DRINK	70	7.54	29.54
2021	DRINK	233	8.79	92.46	CHASE	230	24.23	93.12
	PERSON	221	8.33	87.70	THAT	239	25.18	96.76
	THAT	169	6.37	67.06	THERE	203	21.39	82.19
	CRACKER	164	6.18	65.08	HURRY	155	16.33	62.75
	THERE	164	6.18	65.08	DRINK	80	8.42	32.39

**Table 5:** Number and percent of utterances in each communicative function category.

Category Subcategory	N n	% %
REQUESTS: solicit information, actions, or acknowledgment.	34	25.95
Yes-No questions: solicit the R to affirm, negate, or confirm the P of the S’s U.	1	0.76
Wh-questions: solicit information about the identity, location, or property of an object, event, or situation.	1	0.76
Action requests: solicit R to perform an act.	32	24.43
Permission requests: solicit R to grant permission for S to perform an act.	0	0
Rhetorical questions: solicit R’s acknowledgment for S to continue.	0	0
RESPONSES: directly complement preceding utterances.	41	31.30
Yes-No answers: complement yes-no questions.	2	1.53
Wh-answers: complement Wh-questions.	16	12.21
Agreements: agree with or deny the P of S’s previous U.	12	9.16
Compliances: make explicit compliances with action requests.	5	3.82
Qualifications: qualify, clarify, or otherwise change P of S’s U.	6	4.58
DESCRIPTIONS: represent observable or verifiable aspects of context.	15	11.45
Identifications: label an object, event, person, or situation.	10	7.63
Possessions: indicate who owns or temporarily possesses an object.	0	0
Events: represents the occurrence of an event, action, process, etc.	0	0
Properties: represent characteristics of objects, events, etc.	0	0
Locations: represent location or direction of objects, events, etc.	5	3.82
STATEMENTS: express analytic and institutional facts, beliefs, attitudes, emotions, reasons, etc.	5	3.82
Rules: express conventional procedures, facts, definitions, etc.	0	0
Evaluations: express impressions, attitudes, judgments, etc.	1	0.76
Internal reports: express S’s internal state (emotions, sentiments, sensations)	4	3.05
Attributions: express beliefs about another’s internal state.	0	0
Explanations: report reasons, causes, or motives for acts or predict future states of affairs.	0	0
CONVERSATIONAL DEVICES: regulate contact and conversations.	36	27.48
Boundary markers: initiate or end contact or conversation.	19	14.50
Calls: make contact by soliciting attention.	3	2.29
Accompaniments: signal contact by accompanying S’s action.	7	5.34
Returns: acknowledge, or fill in after, R’s preceding U.	2	1.53
Politeness markers: make explicit S’s politeness.	5	3.82
PERFORMATIVES: accomplish acts by being said.	17	12.98
Role play: establish fantasies.	0	0
Protests: object to R’s previous behavior.	0	0
Jokes: produce humorous effects.	0	0
Game markers: initiate, continue, or end a game.	4	3.05
Claims: establish rights for S by being signed.	0	0
Warnings: alert R of impending harm.	0	0
Teases: annoy, taunt, or provoke R.	0	0
Reassurance: calm, reassure, or provide support to R.	13	9.92
UNINTERPRETABLE: are unintelligible, incomplete, or otherwise incomprehensible utterances.	9	6.87
NO NARRATIVE: insufficient narrative included in sign log.	6	4.58
PRIVATE SIGN—no indication of interaction.	2	1.53

Note: S = Speaker, R = Receiver, P = Proposition.

**Table 6:** Distribution of utterances to human and chimpanzee partners for combined chimpanzees.

	C-H	%	C-C	%
Requests	24	26.97	10	13.70
Responses	40	44.94	0	0.00
Descriptions	14	15.73	1	1.37
Statements	2	2.25	3	4.11
Conversational Devices	6	6.74	30	41.10
Performatives	1	1.12	16	21.92
Uninterpretable	1	1.12	8	10.96

## Data Availability

Data are available with a reasonable request to the corresponding author.
